# Supplementary material for: Efficacy of sanitization protocols in removing parasites in vegetables: A protocol for a systematic review with meta-analysis
Source: PLoS One. 2022 May 10;17(5):e0268258. doi: 10.1371/journal.pone.0268258 (PMC9089895; doi:10.1371/journal.pone.0268258)
Supplement: S1 File — (DOCX) [file pone.0268258.s002.docx]

| SEARCH STRATEGIES | |
| --- | --- |
| Databases | **Search strategy** |
| Ovid MEDLINE | \| 1 \| exp Lettuce/ \| \| --- \| --- \| \| 2 \| vegetables.mp. \| \| 3 \| fresh produce.mp. \| \| 4 \| Plants, Edible.mp. \| \| 5 \| leafy vegetables.mp. \| \| 6 \| 1 or 2 or 3 or 4 or 5 \| \| 7 \| Parasites/ or parasit*.mp. \| \| 8 \| exp Oocysts/ \| \| 9 \| exp Helminths/ \| \| 10 \| Parasite Egg Count/ \| \| 11 \| parasite examination.mp. \| \| 12 \| intestine parasite.mp. \| \| 13 \| 7 or 8 or 9 or 10 or 11 or 12 \| \| 14 \| exp Disinfection/ \| \| 15 \| hypochlorite.mp. or Hypochlorous Acid/ \| \| 16 \| Peracetic Acid/ \| \| 17 \| Hydrogen Peroxide/ \| \| 18 \| Detergents/ \| \| 19 \| Chlorine/ \| \| 20 \| disinfection agent.mp. \| \| 21 \| Decontamination/ \| \| 22 \| chemical agent.mp. \| \| 23 \| Sanitation/ \| \| 24 \| Anti-Infective Agents/ \| \| 25 \| 14 or 15 or 16 or 17 or 18 or 19 or 20 or 21 or 22 or 23 or 24 \| \| 26 \| 6 and 13 and 25 \| |
| Web of Science | \| 1 \| TS=(VEGETABLES) \| \| --- \| --- \| \| 2 \| TS=(LETTUCE \| \| 3 \| TS=("FRESH PRODUCE") \| \| 4 \| TS=("EDIBLE PLANTS") \| \| 5 \| TS=("LEAFY VEGETABLES") \| \| 6 \| TS=("SALAD*") \| \| 7 \| #1 OR #2 OR #3 OR #4 OR #5 OR #6 \| \| 8 \| TS=("PARASIT*") \| \| 9 \| TS=OOCYST \| \| 10 \| TS = HELMINTH* \| \| 11 \| TS = "EGG COUNT \| \| 12 \| TS = "PARASITE EGG COUNT" \| \| 13 \| TS = "PARASITE EXAMINATION" \| \| 14 \| TS = "PARASITE INTESTINE" \| \| 15 \| TS = "ENTAMOEBA" \| \| 16 \| #8 OR #9 OR #10 OR #11 OR #12 OR #13 OR #14 OR #15 \| \| 17 \| TS = "Disinfection" \| \| 18 \| TS = "hypochlorite" \| \| 19 \| TS = "Hypochlorous Acid" \| \| 20 \| TS = "Peracetic Acid" \| \| 21 \| TS = "Hydrogen Peroxide" \| \| 22 \| TS = "Detergent" \| \| 23 \| TS = "Chlorine" \| \| 24 \| TS = "disinfection agent" \| \| 25 \| TS = "Decontamination" \| \| 26 \| TS = "chemical agent" \| \| 27 \| TS = "Sanitation" \| \| 28 \| TS = "Wash" \| \| 29 \| TS = "Clean*" \| \| 30 \| TS = "Anti-Infective Agent*" \| \| 31 \| TS = "Ozone" \| \| 32 \| TS = "Ultraviolet" \| \| 33 \| TS = "Food safety" \| \| 34 \| TS = "Food quality" \| \| 35 \| TS = "Food analysis" \| \| 36 \| TS = "Food contamination" \| \| 37 \| TS = "Food parasitology" \| \| 38 \| TS = "Disinfectants" \| \| 39 \| #17 OR #18 OR #19 OR #20 OR #21 OR #22 OR #23 OR #24 OR #25 OR #26 OR #27 OR #28 OR #29 OR #30 OR #31 OR #32 OR #33 OR #34 OR #35 OR #36 O #37 OR #38 \| \| 40 \| #7 AND #16 AND #39 \| |
| Embase, Scopus | 'leafy vegetable'/exp OR 'romaine lettuce'/exp OR 'iceberg lettuce'/exp OR 'basil'/exp) AND ('disinfection'/exp OR 'sanitation'/exp OR 'disinfectant agent'/exp OR 'decontamination'/exp OR 'chemical agent'/exp) AND ('food quality' OR 'quality control procedures' OR 'food control' OR 'food analysis' OR 'parasitology' OR 'parasites' OR 'parasites egg count' OR 'parasite examination') |
| FSTA, Lilacs e AGRIS | ("Lettuce" OR "Vegetables" OR "fresh produce" OR " Edible Plants" OR "leafy vegetables" OR "Salads") AND (“Parasites” OR “Parasit*” OR “Oocysts” OR “Helminths” OR “Egg Count” OR “Parasite Egg Count” OR “parasite examination” OR “intestine parasite” OR “Entamoeba”) AND (“Disinfection” OR “hypochlorite” OR “Hypochlorous Acid” OR “Peracetic Acid” OR “Hydrogen Peroxide” OR “Detergents” OR “Chlorine” OR “disinfection agent” OR “Decontamination” OR “chemical agent” OR “Sanitation” OR “Wash” OR “Clean” OR “Anti-Infective Agents” OR “Ozone” OR “Ultraviolet” OR “Food safety” OR “Food quality” OR “Food analysis” OR “Food contamination” OR “Food parasitology” OR “Disinfection”) |
