# Supplementary material for: Efficacy of sanitization protocols in removing parasites in vegetables: A protocol for a systematic review with meta-analysis
Source: PLoS One. 2022 May 10;17(5):e0268258. doi: 10.1371/journal.pone.0268258 (PMC9089895; doi:10.1371/journal.pone.0268258)
Supplement: S2 File — (DOCX) [file pone.0268258.s003.docx]

**Additional file 3: Risk of bias evaluation**

**This table was used in this study for assessing the risk of bias in foods and was adapted from the following methodology:**

Higgins JPT, Sterne JAC, Savović J, Page MJ, Hróbjartsson A, Boutron I, Reeves B, Eldridge S. A revised tool for assessing risk of bias in randomized trials In: Chandler J, McKenzie J, Boutron I, Welch V (editors). Cochrane Methods. Cochrane Database of Systematic Reviews 2016, Issue 10 (Suppl 1). <http://dx.doi.org/10.1002/14651858.CD201601>.

Article: Efficacy of sanitization protocols in the removal of parasites in vegetables: a systematic review with meta-analysis protocol

| **Bias domain** | **Bias Source Description** | **Risk of bias judgment** | **Score** |
| --- | --- | --- | --- |
| Selection bias | No randomization, blinding, or randomised generation of sample's numbers that minimizes the influence of the type of intervention for the analyst | High - Processing describes methodology without randomised sample allocation analysis or not informed | 0 |
|  |  | Low - Mentions randomization or random numbering generation or blinding or drawing or any other method that evidences care to don't influence the sample result. Uses an analysis method that suffers low influence from the tendency of the analyst | 2 |
|  |  | Uncertain - The study contains insufficient information to allow judgment in other categories. | 1 |
| Sampling bias | The outcomes contain low credibility due to an unrepresentative sample size in the analyses performed | High - No statistical calculation of the sample's size that represent a lot. Absence of comparison with other studies that justifies the quantitative adopted. | 0 |
|  |  | Low - Presents statistical calculations or comparisons of the analyzed sample size with samples from other studies, or contains a large sample size in relation to the other selected studies, but does not mention statistical calculations or comparisons. | 2 |
|  |  | Uncertain - The study contains insufficient information to allow judgment in other categories. | 1 |
| Performance bias | The significance of the intervention is low due to the analyses don't have comparisons with control samples or experiments without artificial contamination | High - Does not perform comparison with control sample or artificial contamination or compare between treatments or does not perform any other procedure that standardizes sample contamination for the adequate comparison between the intervention. | 0 |
|  |  | Low - References comparison with control or uses artificial contamination of the sample. | 2 |
|  |  | Uncertain - The study contains insufficient information to allow judgment in other categories. | 1 |

| Deteccion bias | The results may have low credibility due to the use of inadequate or non-validated methodology or that presents a strong risk of bias for the analyst. | | High - Uses non-validated or uninformed methodology or modification of methodology without validation. Uses validated detection method, but has subjective influence of the analyst (e.g., microscopy). | | 0 |  |
| --- | --- | --- | --- | --- | --- | --- |
|  |  |  | Low - Utilizes immunoenzymatic or molecular detection (such as PCR) or automatized method that is independent of the analyst's bias or the result is produced by more than one analyst independently or the study had blinding of the analyzed samples | | 2 |  |
|  |  |  | Uncertain - The study contains insufficient information to allow judgment in other categories. | | 1 |  |
| Publication bias | Presents bias in selective reporting of results or indirect results of intervention in the sample | | High - Presents only positive data from the intervention, without citing cases that didn't have the expected effect. Uses samples from the diagnostic stage (such as the extraction stage) that do not correspond to the post-intervention sanitized food | | 0 |  |
|  |  |  | Low - Describes all outcomes, including expected (positive) and unexpected (null effects) outcomes of the intervention processes. | | 2 |  |
|  |  |  | Uncertain - The study contains insufficient information to allow judgment in other categories. | | 1 |  |
|  |  | |  | |  |  |
|  |  | |  | |  |  |
| **Overall Risk Assessment** | | | |  | |  |
| **RisK** | | **Score** | |  | |  |
| **Low** | | ***7 - 10** | |  | |  |
| **Uncertain** | | ***3 - 6** | |  | |  |
| **High** | | ***0 - 2** | |  | |  |
