# Supplementary material for: Efficacy of sanitization protocols in removing parasites in vegetables: A protocol for a systematic review with meta-analysis
Source: PLoS One. 2022 May 10;17(5):e0268258. doi: 10.1371/journal.pone.0268258 (PMC9089895; doi:10.1371/journal.pone.0268258)
Supplement: Attachment — Submitted filename: Response to Reviewers.docx [file pone.0268258.s005.docx]

**Federal University of Rio Grande do Norte**

**Departament of Pharmacy**

Natal, April 22^th^, 2022

Dear Editor,

Thank you for considering our manuscript entitled ***"Efficacy of sanitization protocols in removing parasites in vegetables: A protocol for a systematic review with meta-analysis"*** for revision. We have made the changes suggested by the reviewers (highlighted in yellow). We hope the revised manuscript is now suitable for publication in PLoS One.

Best regards,

Cristiane Fernandes de Assis

Department of Pharmacy

Federal University of Rio Grande do Norte

Phone: (55 84) 99152-7007

E-mail: cristianeassis@hotmail.com

**Answers to reviewer #1:** Present study describes a systematic review protocol with meta-analysis on evaluating the effectiveness of existing sanitation methods in removing parasites from vegetables it will help researcher to better understanding important of food safety. The authors already answered my questions and revised them in the text. recommend it to publishing.

*Thank you for considering the article for publication.*

**Answers to reviewer #2:** Generally, I want to mention again that the topic is interesting and important. The authors have made major modifications by taking into consideration reviewers comment. Specifically, my concern about the protocol is well addressed.

*Thank you for considering the article for publication.*

**Answers to reviewer #3:** The authors have answered all of my questions and made considerable revisions to the manuscript and can be accepted.

*Thank you for considering the article for publication.*

**Answers to reviewer #4:** The authors improved their MS. Some positions in the refs need revision; see 21, 31, pl also check the international literature if you are missing some original related papers.

*We appreciate the comments and made the proper changes in references.*
